# Diagnostic accuracy of the Eurotest for dementia: a naturalistic, multicenter phase II study

**DOI:** 10.1186/1471-2377-6-15

**Published:** 2006-04-10

**Authors:** Cristobal Carnero-Pardo, Manuel Gurpegui, Emilio Sanchez-Cantalejo, Ana Frank, Santiago Mola, M Sagrario Barquero, M Teresa Montoro-Rios

**Affiliations:** 1 Hospital Universitario Virgen de las Nieves, Granada, Spain; 2 Department of Psychiatry and Institute of Neurosciences, Universidad de Granada, Spain; 3 Escuela Andaluza de Salud Pública, Granada, Spain; 4 Hospital Universitario La Paz, Madrid, Spain; 5 Hospital Vega Baja, Orihuela, Alicante, Spain; 6 Hospital Clínico San Carlos, Madrid, Spain

## Abstract

**Background:**

Available screening tests for dementia are of limited usefulness because they are influenced by the patient's culture and educational level. The Eurotest, an instrument based on the knowledge and handling of money, was designed to overcome these limitations. The objective of this study was to evaluate the diagnostic accuracy of the Eurotest in identifying dementia in customary clinical practice.

**Methods:**

A cross-sectional, multi-center, naturalistic phase II study was conducted. The Eurotest was administered to consecutive patients, older than 60 years, in general neurology clinics. The patients' condition was classified as dementia or no dementia according to DSM-IV diagnostic criteria. We calculated sensitivity (Sn), specificity (Sp) and area under the ROC curves (aROC) with 95% confidence intervals. The influence of social and educational factors on scores was evaluated with multiple linear regression analysis, and the influence of these factors on diagnostic accuracy was evaluated with logistic regression.

**Results:**

Sixteen neurologists recruited a total of 516 participants: 101 with dementia, 380 without dementia, and 35 who were excluded. Of the 481 participants who took the Eurotest, 38.7% were totally or functionally illiterate and 45.5% had received no formal education. Mean time needed to administer the test was 8.2+/-2.0 minutes. The best cut-off point was 20/21, with Sn = 0.91 (0.84–0.96), Sp = 0.82 (0.77–0.85), and aROC = 0.93 (0.91–0.95). Neither the scores on the Eurotest nor its diagnostic accuracy were influenced by social or educational factors.

**Conclusion:**

This naturalistic and pragmatic study shows that the Eurotest is a rapid, simple and useful screening instrument, which is free from educational influences, and has appropriate internal and external validity.

## Background

Dementia is relatively easy to diagnose once the clinical picture has become clear, but it is not so easy to detect in very early stages. The early diagnosis of dementia is difficult because this requires experience and ability; moreover, it is expensive in terms of time and resources. However, early diagnosis is desirable and has advantages for patients, their relatives and society [1]. Currently, although there is no convincing evidence for the application of screening tests to pre-symptomatic persons [2], the use of such tests in individuals with suspected cognitive impairment might be helpful [3].

Screening tests for dementia should satisfy appropriate criteria for applicability (brief, simple, easy to administer) and sound psychometric qualities (reliability and validity) [4]. In addition, the results of these instruments should be independent of the subject's socio-demographic and educational characteristics (including illiteracy) [5], and should be applicable for individuals of any cultural background and sensory condition. The influence of educational factors is especially problematic, since proposed score adjustments do not offset the bias and may introduce problems with validity [6].

The Mini-Mental State Examination (MMSE) [7], the Memory Impairment Screen (MIS) [8], the Seven Minute Screen (7 MS) [9], the Clock-drawing Test (CDT) [10], the Time and Change test (T&C) [11], or the Short Test of Mental Status (STMS) [12] are the screening instruments most commonly used. However, all these tests have important limitations. Some of them (MMSE, 7 MS) are too long for routine use in general medical practice [13,14]; some (MMSE, MIS) are not suitable for persons with illiteracy; some (MMSE, CDT, 7 MS, STMS) include paper and pencil items, and are therefore not appropriate for persons with illiteracy or a low level of education [5,15]; and the T&C has not been thoroughly studied and is of limited usefulness when the prevalence of dementia is low [16]. There are some instruments for detecting dementia among non-European populations with high illiteracy rates, as in India or Nigeria [17], yet they take 20 minutes or longer to administer [18] and none has been adapted for use with European populations.

The Eurotest (see Additional File 1), an adaptation to the euro of the Money Test [19], is a brief screening test which can be used with illiterate persons or people with a very low educational level. It is based on the knowledge and handling of currency coins of legal tender (euros), and can be used without modification in all countries within the Common European Economic Space, where the euro is the common currency. A preliminary study in a convenience population sample found the results to have a high diagnostic accuracy and to be independent from educational factors. A high degree of concurrent validity was also found for other instruments used widely in our setting, such as the MMSE, 7 MS and SPMSQ [20].

The aim of this article is to present the results of the *Trans-Eurotest Study*, a Phase II study [21,22] of this screening test which assessed its diagnostic accuracy for the identification of dementia in a large sample of subjects previously diagnosed in an independent manner according to the Diagnostic and Statistical Manual of Mental Disorders, 4th edition (DSM-IV) criteria [23]. Here we also analyze the influence of educational variables on the results.

## Methods

### Participants

Between April and July, 2004, we enrolled patients who fulfilled the following criteria: 1) age older than 60 years; 2) followed continuously by the researchers who participated in this study for any clinical neurological reason, not limited to cognitive impairment; and 3) had an established clinical and cognitive diagnosis before being assessed using the Eurotest. Each researcher selected participants prospectively following a systematic, predetermined protocol: each participant was chosen on the basis of being the first patient seen by the researcher on any given day and fulfilling the inclusion criteria. Only one participant per day was included by each researcher, and each recruited at least 25 participants; this minimum of 25 was set in order to assure that each sub-sample was representative of the researcher's clinic. Being a phase II study, it was not considered necessary to recruit a prospective cohort of newly referred patients. Patients were excluded if they declined to participate or had previously participated in this study. The patient's assessment included a comprehensive clinical, neuropsychological and functional evaluation, as well as neuro-imaging and laboratory studies, according to recommendations by the Spanish Neurological Society [24]. The assessment instruments were selected by the clinical investigators on the basis of their clinical experience and practice. The patients' condition was classified into dementia (DEM) or no dementia (NoDEM) according to the DSM-IV criteria [23]. The diagnosis of dementia required: multiple cognitive deficits manifested by both memory impairment and other cognitive disturbances; significant functional decline from the previous level of functioning due to those cognitive deficits; and the manifestation of these deficits not exclusively during the course of a delirium.

### Procedure

The *Trans-Eurotest Study *presents a cross-sectional multi-center naturalistic phase II study for diagnostic tests [21,22] carried out in general neurology clinics. The Eurotest was administered at the end of a regular clinical interview, and its score did not influence the previous clinical or cognitive diagnosis. The patient's assessment included a comprehensive clinical and neuropsychological evaluation as well as neuro-imaging and laboratory studies.

The following variables were recorded for all participants: sex, age, literacy [as literate or illiterate (unable to read or write, or able to read or write only with difficulty)], completed primary education (no/yes), frequency with which they handled money (daily/not daily), subjective skills in handling money (poor/good), Global Deterioration Scale (GDS) stage (1: No cognitive decline (CD), normal; 2: very mild CD, subjective complaint of memory deficit, age associated memory impairment; 3: mild CD, mild cognitive impairment; 4: moderate CD, mild dementia; 5: moderately severe CD, moderate dementia; 6: severe CD, moderately severe dementia; and 7: very severe CD, severe dementia) [25], clinical diagnosis, the likelihood of a clinical diagnosis inducing cognitive impairment (no/yes), use of medication that could potentially affect cognitive performance (no/yes), results of the Eurotest and time needed to complete it, and results on a verbal (semantic) fluency test (sVFT; naming animals during one minute).

### The instrument

The Eurotest [20] consists of three parts (see Additional File 1). The first part assesses knowledge of the different coins and bills available. One point is scored for each correct answer, and one point is subtracted for each wrong answer; the final score ranges from 0 to 15 points. The second part assesses the person's performance on five arithmetical tasks of increasing difficulty with 11 coins (counting, making change, adding, dividing by two, and dividing by three). Two points are scored for each correct answer on the first try, and one point is scored for each correct answer on the second try. The maximum time allowed for each task is 1 minute, and the final score ranges from 0 to 10 points. The third part assesses the person's recall of the number and type of coins used before, and the total amount of money involved. The final score ranges from 0 to 10 points. The total overall score on the Eurotest thus ranges from 0 to 35 points.

Between the second and the third part of the test, the sVFT was administered as a distraction task; the score on this test did not count for the final score on the Eurotest.

### Statistical analysis

The results for the two groups were compared with Student's *t *test for continuous variables and the chi-squared (χ^2^) test for categorical variables. Diagnostic accuracy was assessed as area under the receiver operating characteristic (ROC) curve (aROC); the clinical diagnosis of dementia was the external gold standard. Sensitivity (Sn), specificity (Sp), and likelihood ratios were calculated for the total score on the Eurotest; the best cut-off score was identified as that which maximized Sn+Sp. The influence of socio-demographic, educational and clinical variables on the Eurotest score (the dependent variable) was determined by multiple linear regression analysis. The diagnostic accuracy of the Eurotest was analyzed by logistic regression, in which the presence of dementia (*vs *no dementia) was the dependent variable and the result on the Eurotest was the predictive variable. The effects of socio-demographic and educational variables were controlled for by comparing adjusted and non-adjusted models to determine associations with these variables and their possible confounding effects on the diagnostic accuracy. To assess convergent validity, we calculated partial correlations (controlling for socio-demographic and educational variables) between the Eurotest, scores on the sVFT, and GDS stage. The diagnostic accuracy of the Eurotest and the sVFT were contrasted by comparing their aROC from the same patients [26]. All parameters were calculated with their 95% confidence intervals (CI), and all the comparisons were two-tailed with an α error of 0.05. All statistical analyses were performed with the SPSS v.11.5 and MedCalc v.7.0 packages for Windows.

### Ethical and methodological considerations

The study protocol was approved by the Torrecárdenas Hospital Institutional Review Board. Informed consent was obtained from all participants as well from the caregivers of the persons in the dementia group.

Both the Trans-Eurotest Study and this report have been conducted adhering to the STARD recommendations for diagnostic studies [27] (see Additional File 2).

## Results

**Figure 1:**
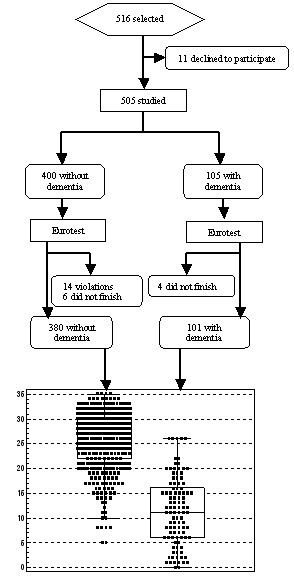
Flow chart of the Trans-Eurotest study and Eurotest scores in patients with and without a diagnosis of dementia

Of the 22 researchers who initially took part in the study; 6 were unable to recruit at least 25 patients, and the results for their 57 patients were not included in the analysis. There were no differences in sex, age or years of clinical experience between researchers who concluded the study and those who did not (data not shown). Among the 16 researchers who concluded the study, the mean number of years of clinical experience was 14.3 ± 8.1 (mean ± sd) (range 4 to 27). These clinicians recruited a total of 516 persons, 11 (2.1%) of whom declined to participate. Ten persons out of 516 (1.9%) did not complete the test (4 because of severe cognitive impairment, and 6 because of sensory or motor impairment), and 14 (2.7%) were excluded because of protocol violations (age younger than 60 years). The final sample thus consisted of 481 persons, 380 with no dementia and 101 with dementia (Figure 1). Of those with dementia, 80% had a diagnosis of Alzheimer's disease.

Among the subjects with no dementia, 197 (51.8%) suffered from a process potentially able to induce cognitive impairment (Parkinson's disease, epilepsy, cerebrovascular disease), 107 (29,1%) expressed complaints of subjective loss of memory (GDS stage 2), 43 (11.3%) were taking medication which could alter cognitive performance, and 79 (20.8%) had some degree of cognitive impairment but did not fulfill the criteria for dementia. About half (54.5%) of the participants with no dementia had at least one of the conditions listed above, and 23.1% had more than one of these conditions.

The characteristics for the sample as a whole are shown in Table 1: mean age was 72.0 ± 6.9 years, women (53.4%) slightly outnumbered men, and level of education was generally low (33.9% were illiterate, and 45.5% had not completed primary school). About one fifth of the participants (21%) did not handle money on a daily basis, and 38.5% felt they did not handle money well. Patients with dementia were significantly older, and the proportion of women, illiterate persons and persons with no formal education was larger in this group than in the group without dementia. A larger number of participants with dementia did not handle money on a daily basis, and felt they did not handle money well. The difference between groups in Eurotest score and sVFT score was highly significant, with worse scores in the group with dementia. Participants in the group with no dementia needed less time to complete the Eurotest (8.0 ± 2.0 minutes) than those in the group with dementia (9.2 ± 2.6 minutes).

In the multiple linear regression analysis, a higher score on the Eurotest was associated with lower GDS stage (β coefficient ± s.e. -3.66 ± 0.19; p < 0.001), younger age (-0.21 ± 0.03; p < 0.001), male sex (-2.07 ± 0.45; p < 0.001), skilful handling of money (1.53 ± 0.52; p < 0.004) and daily use of money (3.48 ± 0.63; p < 0.001); but was not influenced by literacy (0.81 ± 0.61; p = n.s.) or having completed primary education (0.47 ± 0.57; p = n.s.). The daily use of money was not associated to the educational level (r = 0.05, n.s.) or the years of formal education (r = 0.06, n.s.) and was weakly associated to illiteracy (r = 0.14, p < 0.05).

**Figure 2:**
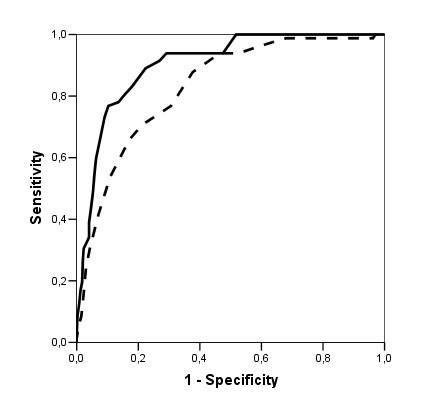
ROC curve as a measure of diagnostic accuracy of the Eurotest (solid line) and the Verbal Fluency Test (dashed line) in identifying dementia

Table 2 shows the distribution of the sample and the values of Sn and Sp for the best cut-offs. The cut-off score that yielded the highest Sn+Sp was 20/21, with Sn = 0.91 (0.84–0.96), Sp = 0.82 (0.77–0.95), LR+ = 5.06 and LR- = 0.11. The aROC of the Eurotest was 0.93 (0.91–0.95) and the aROC of the sVFT was 0.86 (0.83–0.90); these values were significantly different (d = 0.07 ± 0.02, p < 0.001, Figure 2). In Table 3, we report the score-specific likelihood ratios and the probabilities of dementia associated to them for different theoretical dementia prevalences.

With a cut-off score of 20/21, the Eurotest correctly classified 89% of our patients as belonging to the group with or without dementia in the crude logistic regression model, and 90% were correctly classified after adjustment for demographic and educational variables; these percentages were not significantly different (Table 4). The Eurotest score correlated significantly with both the GDS stage (r = -0.72, p < 0.001) and the sVFT score (r = 0.47; p < 0.001).

When the results for the 77 patients with cognitive impairment without dementia are excluded, the aROC increases to 0.96 and the best cut-off score increases to 22/23 with S = 0.95 and Sp = 0.85, which are similar to the values obtained in a preliminary study with a convenience sample [20]. The analysis of the diagnostic accuracy of the Eurotest for cognitive impairment will be reported elsewhere.

Our results for diagnostic accuracy were consistent and stable in different subset analyses. We found no differences in diagnostic accuracy between geographical regions: persons in southern Spain, where levels of education are generally lower than in the rest of the country, scored the same as patients in northern Spain. No differences were seen between the results obtained by clinicians with different levels of experience using the Eurotest; the results were similar for patients who were given the test near the start of the study and for those who took the test near the end of the study. Moreover, the findings were not affected by excluding the data contributed by researchers at the coordinating center in Almería (data not shown).

## Discussion

### Applicability and validity of the Eurotest

The results of the Trans-Eurotest Study show that the Eurotest is rapid and simple to administer, and is a useful, valid instrument that can be applied to patients who are illiterate. The diagnostic accuracy of the Eurotest is not influenced by socio-demographic or educational characteristics. The external validity of these conclusions is supported by the nature and design of the present study. The manner in which participants were recruited, the lenient inclusion criteria, the lack of exclusion criteria and the low rate of refusal to participate speak in favor of the study's pragmatic and naturalistic nature. In fact, more than half of the participants initially included had characteristics that would have made them ineligible for some related studies (e.g., conditions potentially able to induce cognitive impairment, use of medication potentially able to affect cognitive performance, or cognitive impairment without dementia). Moreover, the varied distribution of patients across different GDS stages is further assurance that the full spectrum of cognitive impairment was represented in our sample, from normality to severe dementia, including intermediate stages, particularly those representing cognitive impairment without dementia or subjective complaints of loss of memory. The participation of multiple centers and the number and varied backgrounds of the participating researchers further ensure that the spectrum of professional neurologists practicing in Spain was broadly represented. Thus the characteristics of the study, the researchers, and the patients guarantee the study's external validity, and thus the generalization of our results [28].

Scarcely 2% of the patients in this study were unable to complete the Eurotest in the time allowed. Because the test evaluated performance on day-to-day tasks that patients are usually familiar with, the test was readily accepted by most patients including those who were illiterate or whose level of education was very low. This fact contrasts with tests that involve paper-and-pencil tasks such as the Clock Test. Although these tasks can in theory be completed by illiterate persons, they are not well accepted by them [15]. Another feature that makes the Eurotest simple to administer is that, unlike most other instruments recommended by the AAN, it requires no cards, pictures or other objects, and no record sheets -only coins. With practice, it can be administered without a score sheet, making the Eurotest useful for patients who are hospitalized or who are otherwise unable to come to the neurology clinic. The Eurotest is short and requires less time than the 7 MS [14] or the MMSE [13]. Although the difference in time needed to complete the test differed significantly between persons with and without dementia, this difference was not relevant in practical terms.

The results of the Eurotest are not influenced by socio-demographic or educational variables such as level of literacy, or level of education. This is a major advantage over other available instruments, as the scores do not need to be adjustment for these variables. Moreover, neither the socio-demographic nor the educational factors improve the ability of the test to discriminate between patients with and without dementia.

Few instruments can document this independence from socio-demographic factors. The recently-described Prueba Cognitiva Leganés (PCL), a test that can be given to illiterate persons and whose results are not influenced by educational level, required much longer to complete (11.5 ± 3.2 minutes) despite the fact that 28.8% of the sample was excluded because of impairments potentially able to interfere with the test [29]. In contrast, the Eurotest took much less time to complete (8.2 ± 2.0 minutes) despite the fact that we excluded none of the patients because of cognitive impairment, and only 1.9% failed to complete the test because of cognitive limitations. The results of the MIS are not influenced by educational level [8], but this instrument cannot be used with illiterate persons, and has the further drawback of evaluating only memory. The diagnostic accuracy of the Eurotest was better that that of the sVFT in the same sample of patients and within the range of accuracy values found for other widely-used tests in other samples [20], although direct comparison of these findings with the present results would be inappropriate. It should be emphasized that the Eurotest achieved good diagnostic accuracy despite the fact that our sample of patients included persons who had cognitive impairment without dementia.

However, these results were less clear-cut than those recently reported for the 7 MS [30] and the PCL [29] in a sample of Spanish patients with a low level of education. The reason may lie in the facts that the refusal rate in these two studies, which involved the same sample, was high (27%), and that more than 20% of the participants were excluded because of sensory impairment. These figures contrast with the low rate of refusal to participate in the present study (2.1%), and with the fact that we did not exclude patients because of sensory impairment or for any reason other than refusal to participate. It should nevertheless be noted that these instruments require more than 10 minutes to administer, and are much more complex to administer and to score.

The structure of the Eurotest, which includes items intended to evaluate knowledge, calculation ability and recall, ensures appropriate content validity and face validity. The test also evaluates money handling ability, an important aspect of the patient's functional capacity. Although deterioration of money handling ability is a criterion in most universally accepted instruments used to diagnose dementia, this skill has not previously been included in any screening test. Ecological validity of the Eurotest is ensured by the every-day nature of the tasks and materials, which avoid making patients feel patronized, embarrassed or apprehensive. Adequate construct validity is ensured by the significant correlation between the Eurotest score and the GDS, a measure of the severity of deterioration that covers the full spectrum of cognitive impairment from normality to advanced dementia. Further evidence of construct validity is the correlation between the Eurotest scores and the sVFT, a widely used screening test.

### Strength and limitations of the study

Among the strengths of this study are its sample size, external validity, consistency in the findings, and the fact that the diagnoses had been established previously and were not influenced by the test results. However, a few weaknesses of the study should be pointed out. The participation of many different researchers may have led to differences in how the diagnostic criteria were interpreted, and to possible misclassification bias. This source of bias was probably mitigated by the facts that all researchers were highly experienced practitioners, and that all based their diagnoses on a comprehensive clinical and neuropsychological evaluation, neuro-imaging and laboratory studies, and other widely used and generally accepted criteria. This limitation could in fact be considered a strength if we consider that the diagnoses used as the gold standard were those which were actually on record for the patients. Our patients, therefore, were exposed to all medical (treatment) and social consequences (restriction, protection, etc.) arising from their diagnosis, a fact that no doubt consequently lent a high degree of "consequential validity" [31]. Some of the patients with dementia had very recently been diagnosed whereas others had been treated over different periods of time, but the information on the duration of cognitive complaints or impairments was not recorded; we acknowledge the absence of this aspect of the representativeness of the cohort.

It may be argued as a limitation that the euro currency has been in use only since January 2001, 3 years before this study was carried out, and some non-demented patients had possibly not yet achieved a skillful handling of the new currency leading to some false positive results. According to our clinical experience this is very unusual; moreover, the fact of not acquiring such skills over that time period may reflect some cognitive impairment interfering with learning. Nevertheless, the false positive cases, if any, would count against the diagnostic accuracy of the Eurotest; over the course of time the number of any false positives would be expected to decrease, meaning that further improvements in specificity could be expected.

The brief period between the appearance of the euro as legal tender to the time of the assessment makes it difficult to determine whether the lower scores obtained by persons with dementia were the result of loss of skills possibly acquired shortly after introduction of the common currency, or whether the ability of some patients to learn the new currency was limited by impairments already present in 2001. This distinction, on which the present study sheds no light, may have important implications for the future usefulness of the Eurotest. Recent observations have suggested that financial abilities are impaired before other abilities are affected [32-34], so that testing money handling skills may be useful for the early detection of cognitive impairment or dementia.

The cross-sectional nature of a phase II study, such as the present study, did not allow us to evaluate the predictive power of the Eurotest. A further important limitation is the fact that the researchers who administered the test were not blinded to the patients' clinical diagnosis, and this may have biased how the test was scored. These limitations are characteristic of phase II studies of diagnostic instruments. Phase III studies should be planned so that the predictive power of the Eurotest can be accurately determined in prospective, independent studies in which experimenters are blind to the patients' diagnosis [21,22,35].

## Conclusion

The Eurotest is an easily applicable, useful and valid screening instrument for detecting dementia by assessing money handling ability, an important aspect of the patient's daily living capacity. The present study shows that it is not influenced by socio-demographic or educational characteristics and that it has a good diagnostic accuracy and appropriate content, face and construct validity.

A further advantage of the Eurotest is that it can be used with no modification in any country that uses the euro as currency. This makes the Eurotest applicable for a total population of more than 300 million persons. The test can easily be adapted to any other currency, and equivalent versions of the test are now being evaluated in other countries.

## Competing interests

C. Carnero-Pardo is the creator of the Eurotest. Some of the authors have received honoraries for lecturing for pharmaceutical companies, but in all cases for less than 10,000 €.

## Authors' contributions

C Carnero-Pardo formulated the study design, led data collection, did the analyses, interpreted the results and wrote the first draft of the manuscript. M Gurpegui helped with the interpretation of the results and edited the final version of the manuscript. E Sánchez-Cantalejo helped with data analysis and interpretation of the results, and approved the final version of the manuscript. A Frank, S Mola, M S Barquero and M T Montoro-Ríos discussed the study design, collected data, commented on the results and approved the final version of the manuscript.

## The Trans-Eurotest group

C Carnero-Pardo (Director of the study, Hospital Virgen de las Nieves, Granada, Spain), T García López, P Guardado Santervás, J Rubí Callejón, G Alonso Verdegay (Hospital Torrecárdenas, Almería, Spain); C Creus, M J Pérez-Navarro, P del Saz, I Feria, L Montiel, M T Montoro-Ríos (Hospital Virgen de las Nieves, Granada); A Frank, M Lara, A Miralles (Hospital Universitario La Paz, Madrid, Spain); M S Barquero (Hospital Clínico Universitario San Carlos, Madrid, Spain), S Cousido (Hospital Puerta del Mar, Cádiz, Spain), J L Dobato (Fundación Hospital de Móstoles, Madrid, Spain), M L García de la Rocha (Hospital Central de la Defensa, Madrid, Spain), F Garzón (Hospital Virgen de la Victoria, Málaga, Spain), A Gómez Camello (Hospital Clínico Universitario San Cecilio, Granada, Spain), M Goñi Imízcoz (Hospital Divino Vallés, Burgos, Spain), M Gurpegui (Department of Psychiatry and Institute of Neurosciences, Universidad de Granada, Granada, Spain), B Indakoetxea (Hospital Donosti, San Sebastián, Spain), F Lacruz (Hospital de Navarra, Pamplona, Navarra, Spain), J M Manubens**† **(Hospital Virgen del Camino, Pamplona, Navarra, Spain), S Mola (Hospital Vega Baja, Orihuela, Alicante, Spain), P Regato (Centro de Salud Leganés, Madrid, Spain), and E Sánchez-Cantalejo (Escuela Andaluza de Salud Pública, Granada, Spain).

## Pre-publication history

The pre-publication history for this paper can be accessed here:



## Supplementary Material

Additional File 1 Eurotest Click here for file

Additional File 2 STARD checklist Click here for file

## Figures and Tables

**Table 1:** Demographic, educational, and cognitive test variables according to the presence or absence of dementia

	**Total (n = 481)**	**Patients with dementia (n = 101)**	**Patients with no dementia (n = 380)**	**p**
**Age, years (SD)**	72.0 (6.9)	75.5 (6.3)	71.1 (6.8)	< 0.001
				
**Sex**				0.02
**Men**	224 (46.6%)	37 (36.6%)	187 (49.2%)	
**Women**	257 (53.4%)	64 (63.4%)	193 (50.8%)	
**Completed primary education**	272 (54.5%)	50 (49.5%)	212 (55.8%)	0.16
**Literacy**	318 (66.1%)	58 (57.4%)	260 (68.4%)	0.03
**Daily use of currency**	380 (79.0%)	55 (54.5%)	325 (65.5%)	< 0.001
**Good handling of currency**	296 (61.5%)	36 (35.6%)	260 (68.4%)	< 0.001
GDS*				< 0.001
**Stage 1**	216 (44.9%)		216 (58.7%)	
**Stage 2**	107 (22.2%)		107 (29.1%)	
**Stage 3**	64 (13.3%)	19 (18.8%)	45 (12.2%)	
**Stage 4**	58 (12.1%)	58 (57.4%)		
**Stage 5–7**	24 (4.9%)	24 (23.7%)		
**sVFT score (SD)**	13.5 (5.7)	8.2 (3.8)	14.9 (5.3)	< 0.001
				
**Eurotest**				
**Score (SD)**	22.5 (8.3)	11.5 (6.9)	25.5 (5.7)	< 0.001
**Duration, min (SD)**	8.2 (2.0)	9.2 (2.6)	8.0 (2.5)^#^	< 0.001

sVFT, Verbal Fluency Test (semantic). GDS, Global Deterioration Scale.

*GDS reported in 469 subjects. ^# ^Time measured in 378 subjects.

**Table 2:** Distribution of the subjects according to the results, and diagnostic utility of the Eurotest fordementia

**Eurotest score**	**Patients with dementia**	**Patients with no dementia**	**Sensitivity**	**Specificity**	**LR+**	**LR-**
≤ **16**	76	28	0.75	0.93	10.71	0.27
**17**	4	10	0.79	0.90	7.90	0.23
**18**	3	7	0.82	0.88	6.83	0.20
**19**	3	8	0.85	0.86	6.07	0.17
**20**	**6**	**17**	**0.91**	**0.82**	**5.06**	**0.11**

**21**	2	18	0.93	0.77	4.04	0.09
**22**	2	9	0.95	0.75	3.80	0.07
**23**	5	283	0.95	0.70	3.17	0.07
**Total**	101	380				

LR, likelihood ratio

**Table 3:** Score-specific likelihood ratios of Eurotest and post-test probabilities of dementia associated with different prevalences of dementia

**Eurotest score**	Patients with dementia*	Patients with no dementia*	**LR**	**Prevalence of dementia**				
				
				**0.05**	**0.1**	**0.2**	**0.27**^#^	**0.5**
**≤ 16**	**76 **(0.75)	**28 **(0.07)	**10.7**	0.35	0.54	0.73	0.8	0.91
**17–18**	**7 **(0.07)	**17 **(0.04)	**2.8**	0.08	0.24	0.41	0.51	0.74
**19–20**	**9 **(0.09)	**25 **(0.06)	**1.5**	0.07	0.14	0.27	0.36	0.60
**21–22**	**4 **(0.04)	**27 **(0.07)	**0.6**	0.03	0.06	0.13	0.17	0.37
**≥ 23**	**5 **(0.05)	**283 **(0.74)	**0.07**	<0.01	<0.01	0.02	0.02	0.06
**Total**	**101 **(1.00)	**380 **(1.00)						

LR, likelihood ratio.

*Number of subjects (probability within the column). ^#^Prevalence in this study.

**Table 4:** Logistic regression models for the diagnosis of dementia from the Eurotest results

**Model**	β	**SE**	**OR (95% CI)**	**p**
**Univariate**				
Constant	-2.34			
Eurotest*	-0.28	0.03	0.75 (0.71–0.80)	<0.001
				
**Adjusted**				
Constant	-2.70			
Eurotest*	-0.31	0.03	0.74 (0.69–0.78)	<0.001
Sex	-0.12	0.34	0.89 (0.45–1.76)	0.74
Age*	-0.01	0.03	0.98 (0.93–1.04)	0.59
Literacy	0.53	0.46	1.75 (0.70–4.14)	0.24
Completed primary education	0.36	0.24	1.43 (0.62–3.29)	0.40

β, β Coefficient; SE, standard error; OR (95% CI), odds ratio (95% confidence interval).

*Variables centered on their mean
