# Supplementary material for: Diagnostic accuracy of the Eurotest for dementia: a naturalistic, multicenter phase II study
Source: BMC Neurol. 2006 Apr 10;6:15. doi: 10.1186/1471-2377-6-15 (PMC1459198; doi:10.1186/1471-2377-6-15)
Supplement: Additional File 1 — Eurotest [file 1471-2377-6-15-S1.PDF]

EUROTEST

1.- Part One (Knowledge/Denomination)

"Do you remember what amounts of currency in coin there are nowadays?; Note that the question is about coins, not bills." (Maximum one minute)<br>Cents: ☐ 1 ☐ 2 ☐ 5 ☐ 10 ☐ 20 ☐ 50 Euros ☐ 1 ☐ 2<br>☐ Others (specify): Total correct: Errors:			1
"Do you remember what amounts there are in bills?". (Maximum one minute)<br>Euros: ☐ 5 ☐ 10 ☐ 20 ☐ 50 ☐ 100 ☐ 200 ☐ 500<br>☐ Others (specify): Total correct: Errors:			

2.- Part Two (Calculation)

Place the coins in font of the subject (**2 1-euro, 1 50-cent, 3 20-cent and 5 10-cent coins**) and ask him or her to perform the following tasks consecutively. In the case that the answer is wrong, let the subject know so and allow him/her a second attempt. Some items have more than one valid solution. Allow a maximum of one minute for each item, and go on to the next if there is no correct answer after the time allowed or if both tries are wrong.

"How many coins are there here?" **(11)**<br>☐ Correct ☐ Correct second attempt ☐ Incorrect			
"Will you exchange this coin for the same amount in smaller change?"<br>(Take away one of the two 1 euro coins and show the subject only one euro coin.)<br>☐ Correct ☐ Correct second attempt ☐ Incorrect			
"How much money is there here altogether?" **(3.60 euros)** (Show all the money.)<br>☐ Correct ☐ Correct second attempt ☐ Incorrect			2
"Please divide this money into two equal piles." **(1.80 euros)**<br>☐ Correct ☐ Correct second attempt ☐ Incorrect			
"Please divide this money into three equal piles." **(1.20 euros)**<br>☐ Correct ☐ Correct second attempt ☐ Incorrect			

3.- Distraction Task: Semantic Verbal Fluency

"I would like you to name all the animals that you can think of, whether they live on the land, in the sea, in the air, in the forest, in the farm, or in the house. Please name any and all that come to mind.	

4.- Part Three (Memory)

"To finish up, I´d like you to make a final effort and try to remember:"

"How many coins did I show you before?" **(11)**<br>Answer: ☐ Correct ☐ Incorrect			
"In total how much money was there?" **(3.60 euros)**<br>Answer: ☐ Correct ☐ Incorrect			
"Do you remember exactly what coins there were?"			3

Quantity	Coins			Errors		
☐ __ 5	**10 cent**	__☐	**Total**	**1 cent**	__☐	**Total**
☐ __ 3	**20 cent**	__☐	**Correct**	**2 cents**	__☐	**Errors**
☐ __ 1	**50 cent**	__☐		**5 cents**	__☐	
☐ __ 2	**1 euro**	__☐	____	**2 euros**	__☐	____

TOTAL (1 + 2 + 3)

Observations:
